# Protocol for a mixed-methods evaluation of a massive open online course on real world evidence

**DOI:** 10.1136/bmjopen-2018-025188

**Published:** 2018-08-13

**Authors:** Edward Meinert, Abrar Alturkistani, David Brindley, Alison Carter, Glenn Wells, Josip Car

**Affiliations:** 1 Department of Paediatrics, University of Oxford, Oxford, UK; 2 Department of Public Health and Primary Care, School of Public Health, Imperial College London, London, UK; 3 Department of Infectious Disease Epidemiology, Faculty of Medicine, School of Public Health, Imperial College London, London, UK; 4 Oxford Academic Health Science Centre, Oxford, UK

**Keywords:** massive open online course, real world data, real world evidence, continuing education, data science, information science

## Abstract

**Introduction:**

Increasing number of Massive Open Online Courses (MOOCs) are being used to train learners at scale in various healthcare-related skills. However, many challenges in course delivery require further understanding, for example, factors exploring the reasons for high MOOC dropout rates, recorded low social interaction between learners and the lack of understanding of the impact of a course facilitators’ presence in course engagement. There is a need to generate further evidence to explore these detriments to MOOC course delivery to enable enhanced course learning design. The proposed mixed-methods evaluation of the MOOC was determined based on the MOOC’s aims and objectives and the methodological approaches used to evaluate this type of a course. The MOOC evaluation will help appraise the effectiveness of the MOOC in delivering its intended objectives. This protocol aims to describe the design of a study evaluating learners knowledge, skills and attitudes in a MOOCs about data science for healthcare.

**Methods and analysis:**

Study participants will be recruited from learners who have registered for the MOOC. On registration, learners will be given an opportunity to opt into the study and complete informed consent. Following completion of the course, study participants will be contacted to complete semistructured interviews. Interviews will be transcribed and coded using thematic analysis, with data analysed using two evaluation models: (1) the reach, effectiveness, adoption, implementation, maintenance framework and the (2) Kirkpatrick model drawing data from pre and post-course surveys and post-MOOC semi-structured interviews. The primary goal of the evaluation is to appraise participants' knowledge, skills and attitude after taking the MOOC.

**Ethics and dissemination:**

Ethics approval for this study was obtained from Imperial College London through the Education Ethics Review Process (EERP) (EERP1617-030). A summary of the research findings will be reported through a peer-reviewed journal and will be presented at an international conference.

**Strengths and limitations of this study**

- One strength of the study is the use of qualitative data from semistructured interviews triangulated via evidence experiences to validate reported activity.
- A limitation of the study is the course evaluation may be affected by factors other than the course that may be difficult to identify.
- The evaluation of the course is dependent on participant study recruitment via the course.

## Introduction

Although research about Massive Open Online Courses (MOOCs) have been increasing with their continued and increased popularity, there remain gaps in understanding on how to achieve similar course impact as compared with face-to-face instruction.^1^ There is a need for evidence on MOOCs to determine how to resolve numerous challenges that impede MOOC uptake and completion.^2^ The factors impacting MOOCs delivery can be summarised as (1) low-course completion rates (less than 10%) relative to the number of learners who sign up,^3^ with more recent evaluation of the literature (2017) revealing that high dropout rates remain a challenge for most MOOCs.^1^ (2) Lack of social interaction between learners raises concerns about the open and diverse environments that MOOCs generally should offer.^4^ (3) Not understanding how the role an educator or facilitator in a MOOC plays in promoting interaction and networking for learners.^5^ Further research on how a MOOC can address these issues is needed to facilitate more effective MOOC course design in the future.

Evaluating MOOCs to test their effectiveness is essential for the effective delivery of learning and for appraising its performance. This study will use a mixed methods combining the reach, effectiveness, adoption, implementation, maintenance (RE-AIM) framework and the Kirkpatrick evaluation models to evaluate learners’ acquisition of learning and skills post-MOOC and their attitudes towards the course. This evaluation will be of value to all stakeholders involved including the learning institution that developed the course (Imperial College and the University of Oxford), other researchers and learners. The value of the outcomes of the research is understanding how successful the MOOC was to achieving impact to justify further continuing the MOOC and enhancing delivery. Gathering first-hand information on how participants valued the MOOC, their reasons for undertaking the MOOC, and the perceived impact it has had on their studies, working environment or professional practice, is key in understanding if the MOOC had an impact on participants and is justified for further investment. The value for other researchers will stem from the addition to the literature about MOOCs and MOOC evaluations. The value to students result from the evaluation being used to improve the redesign of the current MOOC and potential improvement of other MOOCs in general based on the insights from this study.

Increased use of data analytics can significantly improve quality and value of health services through increased efficiency and effectiveness.^6^ For example, the use of aggregate population level data collection, as implemented in Real World Data (RWD) approaches, could contribute to advancing capabilities in personalising care, through adjustments in interventions based on real-time analysis of patient responses^7^ and could develop predictive capabilities that can help identify patients at higher risk and contribute to adverse events.^8^ For organisations to receive the full benefits of data analytics, there is a growing demand for training staff in data analysis and ‘to equip managers and employees with relevant professional competencies.’^8^ MOOCs have been successful in delivering new skills. For example, a MOOC on antimicrobial stewardship in low-income and middle-income countries reported that 49% of the participants interviewed 6 months after the MOOC (n=409) have assured that they have implemented the interventions learnt from the MOOC in their practice.^9^ In addition, a randomised controlled trial found that both MOOCs and a self-paced online educational module were useful in training physiotherapists about spinal cord injuries and increasing their confidence about administering therapy to patients.^10^ MOOCs are seen as a suitable method for delivering continuing education in improving their patient care.^11^

This study examines a MOOC centred on Real World Evidence (RWE). The objective of the MOOC is to introduce learners to data analysis methods and techniques of RWE. The MOOC aims to raise awareness of the potential impact RWD data science methods can have on medicine. To evaluate the success of achieving the MOOCs objectives, this study will evaluate the MOOC’s ‘reach’ of its intended audience and social networks, ‘efficacy’ regarding the knowledge/skill gain and attrition, adoption and sustainability of social networks for continual learning in this emerging field; further details of the course instructional design are defined in a separate publication.^12^ The evaluation is conducted not only to contribute to the current literature, but also to enable evidence to support future iterations of the course to increase its impact.

### Research question

The primary research question of the evaluation is: How has the course impacted learners’ knowledge, skills and attitudes on the use of data science in healthcare?

The secondary questions of the evaluation include the following:

- What evidence is there that the intended target audience was reached?
- What evidence is there that the MOOC has made a difference to participants in their work or studies
- What evidence is there of participant networks for data science in healthcare being adopted during the MOOC?
- What evidence is there that the MOOC format and materials engaged participants?
- What evidence is there of participant networks for data science in healthcare being sustained post-­MOOC?

## Methods and analysis

Study participants will be recruited from learners who have registered for the MOOC. On registration to the course, learners will be given an opportunity to opt into the study, receiving a participant information sheet (online Supplementary appendix 1). Study participants will also be recruited via email (online Supplementary appendix 2). Should learners wish to participate, they will sign an informed consent form (online Supplementary appendix 3). Following completion of the course, study participants will be contacted to schedule interviews. A researcher holding postgraduate level training in qualitative research methods (via Imperial College’s Master of Public Health and/or Doctorate training programme in Clinical Medicine Research) will hold semistructured interviews with the study participant (online Supplementary appendix 4).

10.1136/bmjopen-2018-025188.supp1 Supplementary data

10.1136/bmjopen-2018-025188.supp2 Supplementary data

10.1136/bmjopen-2018-025188.supp3 Supplementary data

10.1136/bmjopen-2018-025188.supp4 Supplementary data

### Study design

This study will apply two evaluation methods to investigate the impact of the MOOC. The RE­-AIM framework will be used to evaluate the reach, delivery (implementation) and sustainability (maintenance) of the MOOC with efficacy and adoption examined by the Kirkpatrick model. The Kirkpatrick evaluation will follow the four levels of assessment: reaction, learning, behaviour and results,^13^ where a particular focus will be given to determine if participants were able to increase their learning through the course, if they were able to apply the skills learnt in the course in their study/workplace (adoption) and if through attending the MOOC they were able to influence their broader community (efficacy). The RE­-AIM framework will be used for evaluation of reach at the participant level. We will examine total recruitment on the course and compare their characteristics to eligibility criteria, demographic information and other measures. Facilitators and barriers to individual patient recruitment and suggestions for improvement will be identified through interviews with the research team. Evaluation of the implementation will be done at participant level via a post-course survey (for graded feedback on course delivery to include materials, content, layout and format, etc) and post­-course interviews to discuss perceptions of participants in greater depth. Maintenance will be evaluated at participant level to measure the continuation of MOOC effects over time. This will be conducted via a post­-course interview, held 3 months post-course to identifyspecific examples and evidence to substantiate participants views/claims. To evaluate impact further, the Kirkpatrick evaluation model shall be used. For Level 1 (Reaction), the survey material completed pre-course and post-course will be captured. For learners who did not complete immediate post-­course surveys, this will be noted, but reflections of the course will be captured in the interview (for recording purposes). For Level 2 (learning), the learning record from the MOOC shall be used. For Level 3 (behaviour), the semistructured interviews will investigate the impact of the MOOC on multiple factors of professional behaviour. For Level 4 (results), through aggregation and coding of the interview results, the research team will analyse the overall impact of the results on training.^13^ The Kirkpatrick model was selected for evaluating the MOOC due to it being directed towards evaluating training programmes designed for professional development training,^13^ and since this MOOC was designed to influence learners' skills and behaviour, this was seen as a suitable model. Also, the model is commonly used for MOOC evaluations as other studies have reported using the Kirkpatrick model in their evaluation methodologies.^14–16^

### Participants

All learners who participated in the MOOC for any length of time will be recruited for the study. Participants recruited will differ due to the diversity of participants joining a MOOC in general. Possible differences will be in the level of data science knowledge and MOOC completion. We will try to reflect this diversity in the participants recruited for the study, by categorising participants and aggregating their results in their response group classification (eg, undergraduates in data science who completed the post-course survey). An additional high-level classification is all learners who (1) completed the pre-course survey, (2) completed the post­ course survey, and (3) completed the certificate track (further categories shall be analysed depending on respondents). The exclusion criteria shall exclude learners who are employed by Imperial College London or are known by the researchers, therefore addressing possible power issues.

### Recruitment

The participants in this MOOC self­-select by registering for the course and participants in this study will be drawn from this pool. All those who have participated in the course will be approached, to prevent participation bias. The learners will be contacted by the research team to participate in the interviews. To avoid conflicts, the exclusion criteria have been designed to avoid any power of coercion from the participants. All learners will be contacted via email twice for participation over a 2­-week period. This method is consistent with previous contact methods during the course and was selected to be non-­intrusive to learners.

### Sample size

We are aiming to recruit 16 learners to the study. We predict that interviews from 16 learners will generate enough data for answering the research questions and fall within the scope of effort allocated for this investigation; additionally, the recent literature suggests that this amount of thematic data will be sufficient for qualitative analysis.^17^ However, if there is a need for further investigation of further phenomena or themes, additional participantswill be interviewed until saturation is reached for all the key themes concerning the study objectives.

### Data collection

#### Pre-course and post-course surveys

Pre-course and post-course surveys are surveys that are administered online and that are accessible to all learners who have participated in the MOOC. The surveys capture the learners' general reaction to the course. Pre-course surveys include questions about reasons for taking the course, preferred learning methods and current knowledge of the topic being taught. The post-course survey gives the learners a chance to provide graded feedback on course delivery to include feedback on the materials provided, the content of the course, and the design of the MOOC (layout and format). The delivery of the post-course survey will be segmented cross-sectionally by learner groups identified from course trends (eg, those who completed the course, those who only participated in part of the course, those who registered but did not complete a significant portion of the course). Survey results will be structured on a Likert scale, using a Kruskal-Wallis test to identify comparison of data between groups. Logistic regression analysis will determine statistically significant results among groups. Reporting structure for the surveys are described in a strengthening the reporting of observational studies (STROBE) statement,^18^ which is detailed in online Supplementary appendix 5.

10.1136/bmjopen-2018-025188.supp5 Supplementary data

#### Semistructured interviews

Interviews are scheduled to take place between 30 and 60 min (maximum). The interviews will be executed at greater than 3 months than the course execution to allow for analysis of the impact of the course has had on behaviour. Interviews will be conducted through Skype and telephone conference calls because course participants are distributed globally, and this is the most accessible means of interviewing participants.^19^ All interviews will be recorded. The reason semistructured interviewing is being used is to investigate further what factors could impact behaviour that is not fully understood at this stage in the study. This will also allow for aggregation of responses and the ability to allow further examination of learners' perspectives on the research question categories. 16 learners will take approximately 160 hours of transcription/analysis to complete with a planned effort of 5 weeks. Interview recordings will be transcribed by an internal third party (an Imperial College staff member trained in transcription) and given to study participants for review for accuracy. Reporting structure for the interviews are reported in the consolidated criterion for reporting qualitative research (COREQ) Statement,^20^ which is detailed in the online Supplementary appendix 6.

10.1136/bmjopen-2018-025188.supp6 Supplementary data

### Study ethics

Anonymisation via a unique ID will be created to protect confidentiality. The primary key between unique ID and participant shall be securely held on a secured drive at Imperial College. Only the research administrators will have access to correlate information to respondents (via a primary key) which will be stored on a secured drive at Imperial College. The reason the primary key is being maintained is in the event of participant wishes to withdraw their data from the study; should a request be received all of their corresponding data and files shall be destroyed. Only the research administrators shall have access to this file. The British Educational Research Association guidelines^21^ have been followed for standards in voluntary informed consent. All participants will receive an information sheet with adequate reading time, and all participants will be asked to sign a written informed consent explaining that all participants have the right to withdraw and remove their data should they decide to even after the interview has been completed. To reimburse participants for their time in participating in the interviews, three response participants will be randomly selected to receive a 40-pound voucher from amazon.co.uk. This study will not include children, vulnerable young people or vulnerable adults. If there are problems raised during the study, this will be escalated to the Head of the Department who will act following discussion with the PI.

### Data analysis

Data analysis will be performed using thematic analysis methods and then evaluate the responses based on the RE-AIM framework and Kirkpatrick evaluation models. The RE-AIM framework has been utilised because of its recognition to identify adoption trends,^22^ while the Kirkpatrick method will form a data set for triangulation. Coding of responses will be completed by an independent review of transcripts (by two members of staff) to ensure consistency in analysis.

### Patient and public involvement

Members of the public informed the development of research questions and study objectives via a workshop held at the European Scientific Institute in July 2017. Learners participating in this study shall complete informed consent (online Supplementary appendix 3) and shall receive a copy of results and publications from this work (online Supplementary appendix 1).

## Ethics and dissemination

Ethics approval for this study was obtained from Imperial College London through the Education Ethics Review Process (EERP) (EERP1617-030). A report summarising the research findings will be published in a peer-reviewed journal. A presentation will be given to a selected audience of health professionals and academics, to include individuals from Imperial College. Findings will also be presented at an international conference.

## Supplementary Material

Reviewer comments

Author's manuscript
