# Supplementary material for: Protocol for a mixed-methods evaluation of a massive open online course on real world evidence
Source: BMJ Open. 2018 Aug 13;8(8):e025188. doi: 10.1136/bmjopen-2018-025188 (PMC6091905; doi:10.1136/bmjopen-2018-025188)
Supplement: Supplementary data [file bmjopen-2018-025188supp001.pdf]

APPENDICES

Appendix 1: Information sheet for participants

INFORMATION SHEET FOR PARTICIPANTS

YOU WILL BE GIVEN A COPY OF THIS INFORMATION SHEET.

Data Science Essentials MOOC Evaluation (in Real World Evidence)

Name of researcher: Josip Car MD PhD

We would like to invite you to participate in this research project. You should only participate if you want to; choosing not to take part will not disadvantage you in any way.

Before you decide whether you want to take part, it is important for you to understand why the research is being done and what your participation will involve. Please take time to read the following information carefully and discuss it with others if you wish. Ask us if there is anything that is not clear or if you would like more information.

1. Project aims and rationale

The aim of this study is to establish the perceived impact this form of course delivery had on developing skills in improving healthcare quality and establishing a community of individuals with similar subject-matter interests. The study is to evaluate and explore the factors that may have influenced change in behaviour in professional skills following completion of the course.

By taking part in the study, you will allow the research team to better understand the effectiveness of the course and make recommendations on how to improve the course in subsequent iterations.

2. Participant inclusion and exclusion criteria

The study aims involve 16 participants who have participated in the Data Science Essentials course in Real World Evidence, but this excludes any individual who is employed by Imperial College London or who has professional/personal relationship with Dr Josip Car, Edward Meinert, Mel Toumazos or Tasnime Osama. Should more than 16 indicate interest in partaking, participants will be selected in order to represent as many different working backgrounds and different levels of course completion as possible.

3. Process of participation

It is up to you to decide whether you want to take part. If you choose to participate, you will be given this information sheet to keep and you will be asked to sign a consent form. If you choose to take part in the study, you are still free to withdraw at any time and without giving a reason, and you will be given the option to have your data destroyed. A decision to withdraw at any time, or a decision not to take part, will not impact you in any way.

You will participate in Skype video conference or telephone conference for 30 to 60 minutes where you will be asked interview questions relating to the course and how it may have impacted your

professional practice since taking the course. The sessions shall be recorded by the researchers investigating the impact of the course, subject to your permission, and transcribed by a 3rd party at Imperial College. If you are a member of NHS staff you will require line management approval to participate in this study.

4. Risks to participants

There is a risk that participants may be impacted negatively in terms of the cost to their time that participation would require, therefore the interviews will be completed in less than 60 minutes. The interviews will be transcribed by an internal third party in Imperial and information will not be disclosed externally.

5. Process for ensuring anonymity and confidentiality

If you consent to take part in the research, any information you provide may be inspected and used by administrators of the study.

Each participant will be anonymised using a unique identifier to maintain confidentiality and all data will be securely stored and managed according to Imperial College rules and expected practices.

Raw, un-anonymised audio data will be securely stored separately from the anonymisation key and deleted when it is no longer needed. The anonymised transcripts will be securely stored according to Imperial College protocols and regulations

6. Access to information

All information which is collected about you during the study will be strictly confidential and accessible only by Imperial College study administrators. Any information which leaves the research administration will have your personal information removed so that you cannot be recognised from it.

7. Possible benefits to participation

By taking part in the study, you will allow the research team to better understand the effectiveness of the course and make recommendations on how to improve the course in subsequent iterations. Additionally, all of those who participate in the interviews will be entered into a prize draw, and three will be randomly selected to receive a £40 (or currency equivalent) Amazon voucher.

8. Plans for dissemination

A report summarising the findings will be published in a peer reviewed journal.

A copy of the study information sheet, along with a copy of the consent form will be provided to you, should you choose to take part in the study.

If you have any questions or require more information about this study, please contact the co-researcher using the following contact details:

Edward Meinert

Global eHealth Unit

Department of Primary Care and Public Health, School of Public Health, Imperial College London

The Reynolds Building | St Dunstan’s Road| London W6 8RP | UK

Email: e.meinert14@imperial.ac.uk | Telephone: +44 (0)782 444 6808.

Thank you for taking the time to read and consider this information.
