# Supplementary material for: Protocol for a mixed-methods evaluation of a massive open online course on real world evidence
Source: BMJ Open. 2018 Aug 13;8(8):e025188. doi: 10.1136/bmjopen-2018-025188 (PMC6091905; doi:10.1136/bmjopen-2018-025188)
Supplement: Supplementary data [file bmjopen-2018-025188supp002.pdf]

Appendix 2: Sample Recruitment Email

Dear Participant,

Thank you for your interest in the Data Science Essentials course in Real World Evidence.

To evaluate the course, we are looking for participants who are interested in having a phone interview or skype discussion about their experience of the MOOC and the effect is has had on their working environment or academic advancement. The interview will take from 30 to 60 minutes. To compensate you for your time, three participants will be randomly entered into a draw to receive a £40 voucher for amazon.co.uk (around a 1 in 5 chance).

If you have any questions, please send them to gehu-training@imperial.ac.uk

If you would like to register your interest, please click the link below:

(link to survey monkey)
