# Supplementary material for: Protocol for a mixed-methods evaluation of a massive open online course on real world evidence
Source: BMJ Open. 2018 Aug 13;8(8):e025188. doi: 10.1136/bmjopen-2018-025188 (PMC6091905; doi:10.1136/bmjopen-2018-025188)
Supplement: Supplementary data [file bmjopen-2018-025188supp003.pdf]

Appendix 3: Informed Consent Form

INFORMED CONSENT FORM

Title of Project: **Data Science Essentials (in Real World Evidence) MOOC Evaluation**

Name of researcher: Josip Car MD PhD

Please initial box.

1. I confirm that I have read and understand the subject information sheet dated 31/06/2017 version 1.0 for the above study. I may keep this information sheet for my records and I have had the opportunity to ask questions which have been answered fully. ☐

2. I understand that my participation is voluntary and I am free to withdraw, without giving any reason and without being penalised or disadvantaged in any way. ☐

3. I understand that sections of my recorded comments and transcript text may be looked at by responsible individuals from Imperial College London. I give permission for these individuals to access this data as relevant to this and future research. ☐

4. I am willing to have this interview recorded. ☐

5. I understand that this consent form will be kept separate from the data and that the researchers will maintain my anonymity throughout the project, including in publication. ☐

6. I confirm that I am not employed by Imperial College London or have a personal/professional relationship with Dr Josip Car, Edward Meinert, Mel Toumazos or Tasnime Osama ☐

7. I agree to take part in the above study. ☐

* *Delete as appropriate*

____________________	____________	____________________
**Name of Participant (Printed)**	**Date**	**Signature**

____________________	____________	____________________
**Name of Researcher**	**Date**	**Signature**

(Printed)

1 copy for subject; 1 copy for researcher.
