# Supplementary material for: Protocol for a mixed-methods evaluation of a massive open online course on real world evidence
Source: BMJ Open. 2018 Aug 13;8(8):e025188. doi: 10.1136/bmjopen-2018-025188 (PMC6091905; doi:10.1136/bmjopen-2018-025188)
Supplement: Supplementary data [file bmjopen-2018-025188supp004.pdf]

Appendix 4: Sample interview questions

- Participant background
  - Current degree course/ working role
  - Education
  - Experience of healthcare
  - Experience of data science methodologies and tools
  - Experience in taking part in MOOCs
  - Experience of using social media.

- Participant's need for a MOOC in Data Science focusing on healthcare data.

  This section will have a series of follow up questions with opportunity for in depth discussion

  - Does this meet a current a need?
  - Have they encountered any barriers to learning in this field?
  - How did this method of delivery (the MOOC) meet their needs?
  - What have they come across which already exists to meet this need?
- Why participant signed-up to the MOOC.

  - The expectations and aims of participants

  This section will cover the expectations of participants when joining the course, and what they aimed to take from it

- How much of MOOC had individual completed?
  Follow up questions:
  - More or less than hoped?
  - What factors helped and hindered this?
  - What modules did they complete?
  - If not finished, did they intend to?

- Social media and creating online learning communities
  Exploring whether the interviewee collaborates and learns via MOOC networks
    - Did they join in during the course? What were their thoughts?
    - Are they still connected with other MOOC learners via networks?
- What did individuals enjoy about the MOOC and why?
    - Views on content, materials and format.

- What could be improved or done differently?
  Follow up question:
    - Any technical issues?

- Value for participant
    - What value did you take from having done the MOOC?
    - How valuable was the networking aspect of the MOOC?
    - Do you have specific examples of data science projects/work that you have implemented or been involved in since undertaking the MOOC?
    - How likely are these projects to have happened if you had not participated in the MOOC?

  Potential follow up:
    - Enquiring as to whether this was in line with their original expectation, and why it may have differed

- Application of learning
    - Have participants been able to apply learning from the MOOC?
    - Do they have plans to apply in the future?
    - How has it affected them in a day-to-day way?

- Are they planning of applying for certification?
  Follow up:
  Why or why not?
