# Supplementary material for: Protocol for a mixed-methods evaluation of a massive open online course on real world evidence
Source: BMJ Open. 2018 Aug 13;8(8):e025188. doi: 10.1136/bmjopen-2018-025188 (PMC6091905; doi:10.1136/bmjopen-2018-025188)
Supplement: Author's manuscript [file bmjopen-2018-025188.draft_revisions.pdf]

BMJ Open is committed to open peer review. As part of this commitment we make the peer review history of every article we publish publicly available.

When an article is published we post the peer reviewers' comments and the authors' responses online. We also post the versions of the paper that were used during peer review. These are the versions that the peer review comments apply to.

The versions of the paper that follow are the versions that were submitted during the peer review process. They are not the versions of record or the final published versions. They should not be cited or distributed as the published version of this manuscript.

BMJ Open is an open access journal and the full, final, typeset and author-corrected version of record of the manuscript is available on our site with no access controls, subscription charges or pay-per-view fees (http://bmjopen.bmj.com).

If you have any questions on BMJ Open's open peer review process please email info.bmjopen@bmj.com

BMJ Open

Protocol for a Mixed-Methods Evaluation of a Massive Open Online Course on Real World Evidence

Journal:	*BMJ Open*
Manuscript ID	bmjopen-2018-025188
Article Type:	Protocol
Date Submitted by the Author:	03-Jul-2018

Complete List of Authors:	Meinert, Edward; Imperial College London, Primary Care and Public Health; University of Oxford, Paediatrics<br>Alturkistani, Abrar; Imperial College London, Primary Care and Public Health<br>Brindley, David; University of Oxford, Paediatrics; University of Oxford, Said Buisness School<br>Carter, Alison; University of Oxford, Paediatrics<br>Wells, Glenn; Oxford Academic Health Science Centre<br>Car, Josip; Imperial College London, Primary Care and Social Medicine

Keywords:	Massive Open Online Course, Real World Data, Real World Evidence, Continuing education, Data science, Information science

SCHOLARONE™
Manuscripts

Protocol for a Mixed-Methods Evaluation of a Massive Open Online Course on Real World Evidence

Edward Meinert, Abrar Alturkistani, David Brindley, Alison Carter, Glenn Wells, Josip Car

Department of Paediatrics, University of Oxford, OX3 9DU Oxford, United Kingdom Edward Meinert Sir David Cooksey Fellow in Healthcare Translation Global eHealth Unit, Department of Public Health and Primary Care, School of Public Health, Imperial College London, W6 8RP London, United Kingdom Edward Meinert Honorary Research Fellow Department of Infectious Disease Epidemiology, Faculty of Medicine, School of Public Health, Imperial College London, W6 8RP London, United Kingdom Abrar Alturkistani Research Assistant Department of Paediatrics, University of Oxford, OX3 9DU Oxford, United Kingdom David Brindley Senior Research Fellow Department of Paediatrics, University of Oxford, OX3 9DU Oxford, United Kingdom Alison Carter Research Associate Oxford Academic Health Science Centre, OX4 4GA Oxford, United Kingdom Glenn Wells Chief Operating Officer Global eHealth Unit, Department of Public Health and Primary Care, School of Public Health, Imperial College London, W6 8RP London, United Kingdom Josip Car Director of the Global eHealth Unit

Correspondence to: Edward Meinert edward.meinert@paediatrics.ox.ac.uk, e.meinert14@imperial.ac.uk 00447824446808

Word Count:

Keywords: Massive Open Online Course; Real World Evidence; Real World Data; Information Science; Continuing education; Data science

For peer review only

ABSTRACT

Introduction

Increasing number of Massive Open Online Courses (MOOCs) are being used to train learners at scale in various healthcare related skills. However, many challenges in course delivery require further understanding, for example, factors exploring the reasons for high MOOC dropout rates, recorded low social interaction between learners and the lack of understanding of the impact of a course facilitators' presence in course engagement. There is a need to generate further evidence to explore these detriments to MOOC course delivery to enable enhanced course learning design. The proposed mixed-methods evaluation of the MOOC was determined based on the MOOC's aims and objectives and the methodological approaches used to evaluate this type of a course. The MOOC evaluation will help appraise the effectiveness of the MOOC in delivering its intended objectives. This protocol aims to describe the design of a study evaluating learners knowledge, skills and attitudes in a Massive Open Online Course (MOOC) about data science for healthcare.

Methods and Analysis

This study will use two evaluation models: 1) The RE-AIM framework and the 2) Kirkpatrick model drawing data from pre and post-course surveys and post-MOOC semi-structured interviews. The primary goal of the evaluation is to appraise participants' knowledge, skills, and attitude after taking the MOOC.

Ethics and dissemination

Ethics approval for this study was obtained from Imperial College London through the Education Ethics Review Process (EERP) (EERP1617-030). A summary of the research findings will be reported through a peer-reviewed journal and will be presented at an international conference.

For peer review only

For peer review only - http://bmjopen.bmj.com/site/about/guidelines.xhtml

STRENGTHS AND LIMITATIONS OF THIS STUDY

- One strength of the study is the use of qualitative data from semi-structured interviews triangulated via evidence experiences to validate reported activity
- A limitation of the study is the course evaluation may be affected by factors other than the course that may be difficult to identify
- The evaluation of the course is dependent on participant study recruitment via the course

For peer review only

INTRODUCTION

Although research about Massive Open Online Courses (MOOCs) have been increasing with their continued and increased popularity, there remain gaps in understanding on how to achieve similar course impact as compared to face to face instruction.[1] There is a need for evidence on MOOCs to determine how to resolve numerous challenges that impede MOOC uptake and completion.[2] The factors impacting MOOCs delivery can be summarised as 1) low course completion rates (less than 10%) relative to the number of learners who sign up,[3] with more recent evaluation of the literature (2017) revealing that high dropout rates remain a challenge for most MOOCs.[1] 2) lack of social interaction between learners raises concerns about the open and diverse environments that MOOCs generally should offer.[4] 3) Not understanding how the role an educator or facilitator in a MOOC plays in promoting interaction and networking for learners.[5] Further research on how a MOOC can address these issues is needed to facilitate more effective MOOC course design in the future.

Evaluating MOOCs to test their effectiveness is essential for the effective delivery of learning and for appraising its performance. This study will use a mixed-methods combining the RE-AIM framework and the Kirkpatrick evaluation models to evaluate learners' acquisition of learning and skills post-MOOC, and their attitudes towards the course. The approach can enhance the quality and the depth of the analysis. [19] This evaluation will be of value to all stakeholders involved including the learning institution that developed the course (Imperial College and the University of Oxford), other researchers and learners. The value of the outcomes of the research is understanding how successful the MOOC was to achieving impact to justify further continuing the MOOC and enhancing delivery. Gathering first-hand information on how participants valued the MOOC, their reasons for undertaking the MOOC, and the perceived impact it has had on their studies or working environment or professional practice since this is key in understanding if the MOOC had an impact on participants and is justified for further investment. The value for other researchers will stem from the addition to the literature about MOOCs and MOOC evaluations. The value to students result from the evaluation being used to improve the redesign of the current MOOC and potential improvement of other MOOCs in general based on the insights from this study.

Increased use of data analytics can significantly improve quality and value of health services through increased efficiency and effectiveness.[6] For example, the use of aggregate population level data collection, as implemented in Real World Data (RWD) approaches, could contribute to advancing

capabilities in personalising care, through adjustments in interventions based on real-time analysis of patient responses,[7] and could develop predictive capabilities that can help identify patients at higher risk and contribute to adverse events.[8] For organisations to receive the full benefits of data analytics, there is a growing demand for training staff in data analysis and "to equip managers and employees with relevant professional competencies."[8] MOOCs have been successful in delivering new skills. For example, a MOOC on antimicrobial stewardship in low and middle-income countries reported that 49% of the participants interviewed six months after the MOOC (n=409) have assured that they have implemented the interventions learned from the MOOC in their practice.[9] In addition, a Randomised Controlled Trial found that both MOOCs and a self-paced online educational module were useful in training physiotherapist about spinal cord injuries and increasing their confidence about administering therapy to patients.[10] MOOCs are seen as a suitable method for delivering continuing education in improving their patient care.[11]

This study examines a MOOC centred on Real World Evidence (RWE). The objective of the MOOC is to introduce learners to data analysis methods and techniques of RWE. The MOOC aims to raise awareness of the potential impact RWD data science methods can have on medicine. To evaluate the success of achieving the MOOCs objectives, this study will evaluate the MOOC's 'reach' of its intended audience and social networks, 'efficacy' regarding the knowledge/skill gain skill and attrition, and adoption and sustainability of social networks for continual learning in this emerging field. The evaluation is conducted not only to contribute to the current literature, but also to enable evidence to support future iterations of the course to increase its impact.

Research Question

The primary research question of the evaluation is: How has the course impacted learners' knowledge, skills and attitudes on the use of data science in healthcare?

The secondary questions of the evaluation include the following:

- What evidence is there that the intended target audience was reached?
- What evidence is there that the MOOC has made a difference to participants in their work or studies
- What evidence is there of participant networks for data science in healthcare being adopted during the MOOC?
- What evidence is there that the MOOC format and materials engaged participants?
- What evidence is there of participant networks for data science in healthcare being sustained post--MOOC?

METHODS AND ANALYSIS

Study Design

This study will apply two evaluation methods to investigate the impact of the MOOC. The RE--AIM framework will be used to evaluate the reach, delivery (implementation) and sustainability (maintenance) of the MOOC with efficacy and adoption examined by the Kirkpatrick Model. The Kirkpatrick evaluation will follow the four levels of assessment: reaction, learning, behaviour and

For peer review only - http://bmjopen.bmj.com/site/about/guidelines.xhtml

results, [12] where a particular focus will be given to determine if participants were able to increase their learning through the course, if they were able to apply the skills learned in the course in their study/workplace (adoption) and if through attending the MOOC they were able to influence their broader community (efficacy). The RE--AIM framework will be used for evaluation of reach at the participant level. We will examine total recruitment on the course and compare their characteristics to eligibility criteria, demographic information and other measures. Facilitators and barriers to individual patient recruitment and suggestions for improvement will be identified through interviews with the research team. Evaluation of the implementation will be done at participant level via a post-course survey (for graded feedback on course delivery to include materials, content, layout and format, etc.) and post-course interviews to discuss perceptions of participants in greater depth. Maintenance will be evaluated at participant level to measure the continuation of MOOC effects over time. This will be conducted via a post-course interview, held three months post-course. For specific examples and evidence to substantiate participants views/claims To evaluate impact further, the Kirkpatrick evaluation model shall be used. For Level 1 (Reaction), the survey material completed pre and post-course will be captured. For learners who did not complete immediate post--course surveys, this will be noted, but reflections of the course will be captured in the interview (for recording purposes). For Level 2 (Learning), the learning record from the MOOC shall be used. For Level 3 (Behaviour), the semi- structured interviews will investigate the impact of the MOOC on multiple factors of professional behaviour. For Level 4 (Results), through aggregation and coding of the interview results, the research team will analyse the overall impact of the results on training. [12] The Kirkpatrick model was selected for evaluating the MOOC due to it being directed towards evaluating training programmes designed for professional development training,[12] and since this MOOC was designed to influence learners' skills and behaviour, this was seen as a suitable model. Also, the model is commonly used for MOOC evaluations as other studies have reported using the Kirkpatrick model in their evaluation methodologies. [13-15]

Participants

All learners who participated in the MOOC for any length of time will be recruited for the study. Participants recruited will differ due to the diversity of participants joining a MOOC in general. Possible differences will be in the level of data science knowledge and MOOC completion. We will try to reflect this diversity in the participants recruited for the study, by categorising participants and aggregating their results in their response group classification (e.g. undergraduates in data science who completed the post-course survey). An additional high-level classification is all learners who (1) - completed the pre-course survey, (2) -completed the post- course survey, (3) completed the certificate track; (further categories shall be analysed depending on respondents). The exclusion criteria shall exclude learners who are employed by Imperial College London or are known by the researchers, therefore addressing possible power issues.

Recruitment

The participants in this MOOC self--select by registering for the course and participants in this study will be drawn from this pool. All those who have participated in the course will be approached, to prevent participation bias. The learners will be contacted by the research team to participate in the interviews. To avoid conflicts, the exclusion criteria have been designed to avoid any power of

For peer review only - http://bmjopen.bmj.com/site/about/guidelines.xhtml

coercion from the participants. All learners will be contacted via email twice for participation over a two--week period. This method is consistent with previous contact methods during the course and was selected to be non-intrusive to learners.

Sample Size

We are aiming to recruit 16 learners to the study. We predict that interviews from 16 learners will generate enough data for answering the research questions and perform the analyses because based on past qualitative interview research review, 16 interviews is within the range of an acceptable number of interviews. [16] However, if there is a need for further investigation of further phenomena or themes, we will endeavour to get more participants into being interviewed for the study. Participants will be interviewed until saturation is reached for all the key themes about course effectiveness.

Data Collection

Pre and post-course surveys

Pre and post-course surveys are surveys that are administered online and that are accessible to all learners who have participated in the MOOC. The surveys capture the learners' general reaction to the course. Pre-course surveys include questions about reasons for taking the course, preferred learning methods, and current knowledge of the topic being taught. The post--course survey gives the learners a chance to provide graded feedback on course delivery to including feedback on the materials provided, the content of the course, and the design of the MOOC (layout and format).

Semi-structured interviews

Interviews are scheduled to take place between 30 to 60 minutes (maximum). The interviews will be executed at greater than three months than the course execution to allow for analysis of the impact of the course has had on behaviour. Interviews will be conducted through Skype and telephone conference calls because course participants are distributed globally, and this is the most accessible means of interviewing participants. [17] All interviews will be recorded. The reason semi-structured interviewing is being used is to investigate further what factors could impact behaviour that is not fully understood at this stage in the study. This will also allow for aggregation of responses and the ability to allow further examination of learners' perspectives on the research question categories. 16 learners will take approx. 160 hours of transcription/analysis to complete with a planned effort of 5 weeks. Interview recordings will be transcribed by an internal third party (an Imperial College staff member trained in transcription).

Study Ethics

Anonymisation via a unique ID will be created to protect confidentiality. The primary key between unique ID and participant shall be securely held on a secured drive at Imperial College. Only the research administrators will have access to correlate information to respondents (via a primary key) which will be stored on a secured drive at Imperial College. The reason the primary key is being maintained is in the event of participant wishes to withdraw their data from the study; should a

For peer review only - http://bmjopen.bmj.com/site/about/guidelines.xhtml

request be received all of their corresponding data and files shall be destroyed. Only the research administrators shall have access to this file. The British Educational Research Association (BERA) guidelines, [18] have been followed for voluntary informed consent use of methods and voluntary informed consent, use of methods and departmental policies in the event there are issues in delivery. All participants will receive an information sheet with adequate reading time, and all participants will be asked to sign a written informed consent explaining that all participants have the right to withdraw and remove their data should they decide to even after the interview has been completed. To reimburse participants for their time in participating in the interviews, three response participants will be randomly selected to receive a 40-pound voucher from amazon.co.uk. This study will not include children, vulnerable young people or vulnerable adults. If there are problems raised during the study, this will be escalated to the Head of the Department who will take action with PI.

Data Analysis

Data analysis will be performed using thematic analysis methods and then evaluate the responses based on the RE-AIM framework and Kirkpatrick evaluation models. Coding of responses will be completed by an independent review of transcripts (by two members of staff) to ensure consistency in analysis.

ETHICS AND DISSEMINATION

Ethics approval for this study was obtained from Imperial College London through the Education Ethics Review Process (EERP) (EERP1617-030). A report summarising the research findings will be published in a peer-reviewed journal. A presentation will be given to a selected audience of health professionals and academics, to include individuals from Imperial College. Findings will also be presented at an international conference.

For peer review only

Contributors

EM conceived the study and drafted the manuscript. JC contributed to the protocol design. All authors read and approved the final manuscript. EM is the guarantor.

Acknowledgements

This work was funded by EIT Health (Grant 18654). This work was also supported by the Sir David Cooksey Fellowship in Healthcare Translation and the SENS Research Foundation.

Conflicts of Interest

The ICMJE uniform disclosure form at http://www.icmje.org/conflicts-of-interest/ was completed by all authors. All authors declare that they have no relevant conflicts of interest, no financial relationships that could potentially influence the submitted manuscript. All authors do not have any patents that may be relevant to the manuscript or any relationship, circumstances or conditions that may lead to conflicts of interest. This article represents the authors' individual opinions and may not necessarily represent the viewpoints of their employers. D.B. is a stockholder in Translation Ventures. Ltd. (Charlbury, Oxfordshire, UK) and IP Asset Ventures Ltd. (Oxford, Oxfordshire, UK), companies that, among other services, provide cell therapy biomanufacturing, regulatory, and financial advice to pharmaceutical clients. D.B. is also subject to the CFS institute's codes, standards, and guidelines, so he must stress that this piece is provided for academic interest only and must not be seen in any way as an investment recommendation.

Funding statement

This work was funded by EIT Health (Grant 18654).

For peer review only

References

1. Bozkurt A, Akgün-Özbek E, Zawacki-Richter O. Trends and Patterns in Massive Open Online Courses: Review and Content Analysis of Research on MOOCs (2008-2015). *The International Review of Research in Open and Distributed Learning* 2017;**18**. doi:10.19173/irrodl.v18i5.3080
2. Sinclair J, Boyatt R, Rocks C, *et al.* Massive open online courses : a review of usage and evaluation. *International journal of learning technology* 2015;**10**:1–23.
3. Khalil H, Ebner M. MOOCs Completion Rates and Possible Methods to Improve Retention - A Literature Review. Association for the Advancement of Computing in Education (AACE) 2014. 1305–13.https://www.learntechlib.org/primary/p/147656/ (accessed 2 Jul 2018).
4. Tawfik AA, Reeves TD, Stich AE, *et al.* The nature and level of learner–learner interaction in a chemistry massive open online course (MOOC). *J Comput High Educ* 2017;**29**:411–31. doi:10.1007/s12528-017-9135-3
5. Goldie JGS. Connectivism: A knowledge learning theory for the digital age? *Med Teach* 2016;**38**:1064–9. doi:10.3109/0142159X.2016.1173661
6. Bates DW, Heitmueller A, Kakad M, *et al.* Why policymakers should care about “big data” in healthcare. *Health Policy and Technology* 2018;**7**:211–6. doi:10.1016/j.hlpt.2018.04.006
7. Firouzi F, Rahmani AM, Mankodiya K, *et al.* Internet-of-Things and big data for smarter healthcare: From device to architecture, applications and analytics. *Future Generation Computer Systems* 2018;**78**:583–6. doi:10.1016/j.future.2017.09.016
8. Wang Y, Kung L, Byrd TA. Big data analytics: Understanding its capabilities and potential benefits for healthcare organizations. *Technological Forecasting and Social Change* 2018;**126**:3–13. doi:10.1016/j.techfore.2015.12.019
9. Sneddon J, Barlow G, Bradley S, *et al.* Development and impact of a massive open online course (MOOC) for antimicrobial stewardship. *J Antimicrob Chemother* 2018;**73**:1091–7. doi:10.1093/jac/dkx493
10. Hossain MS, Shofiqul Islam M, Glinsky JV, *et al.* A massive open online course (MOOC) can be used to teach physiotherapy students about spinal cord injuries: a randomised trial. *Journal of Physiotherapy* 2015;**61**:21–7. doi:10.1016/j.jphys.2014.09.008
11. Pickering JD, Swinnerton BJ. An Anatomy Massive Open Online Course as a Continuing Professional Development Tool for Healthcare Professionals. *MedSciEduc* 2017;**27**:243–52. doi:10.1007/s40670-017-0383-7
12. Kirkpatrick DL. *Evaluating training programs: the four levels*. 3rd ed. San Francisco, CA: Berrett-Koehler 2005.
13. Lin J, Cantoni L. Assessing the Performance of a Tourism MOOC Using the Kirkpatrick Model: A Supplier’s Point of View. *Information and Communication Technologies in Tourism 2017* 2017;:129–42. doi:10.1007/978-3-319-51168-9_10

For peer review only

14. Ayub E, Wei GW, Yue WS. Exploring Factors Affecting Learners' Acceptance of MOOCs Based on Kirkpatrick's Model. In: *Proceedings of the 8th International Conference on E-Education, E-Business, E-Management and E-Learning*. New York, NY, USA: : ACM 2017. 34–39. doi:10.1145/3026480.3026490
15. Goh WW, Wong SY, Ayub E. The Effectiveness of MOOC Among Learners Based on Kirkpatrick's Model. *Redesigning Learning for Greater Social Impact* 2018;:313–23. doi:10.1007/978-981-10-4223-2_29
16. Saunders MNK, Townsend K. Reporting and Justifying the Number of Interview Participants in Organization and Workplace Research. *British Journal of Management*;**27**:836–52. doi:10.1111/1467-8551.12182
17. Janghorban R, Roudsari RL, Taghipour A. Skype interviewing: The new generation of online synchronous interview in qualitative research. *Int J Qual Stud Health Well-being* 2014;**9**. doi:10.3402/qhw.v9.24152
18. British Education Research Association. BERA Ethical Guidelines for Educational Research 2011. Researchers' Resources. 2013.https://www.bera.ac.uk/researchers-resources/publications/bera-ethical-guidelines-for-educational-research-2011 (accessed 2 Jul 2018).
19. Patton MQ. Enhancing the quality and credibility of qualitative analysis. *Health Serv Res* 1999;**34**:1189–208.

For peer review only

BMJ Open

Protocol for a Mixed-Methods Evaluation of a Massive Open Online Course on Real World Evidence

Journal:	*BMJ Open*
Manuscript ID	bmjopen-2018-025188.R1
Article Type:	Protocol
Date Submitted by the Author:	19-Jul-2018

Complete List of Authors:	Meinert, Edward; Imperial College London, Primary Care and Public Health; University of Oxford, Paediatrics<br>Alturkistani, Abrar; Imperial College London, Primary Care and Public Health<br>Brindley, David; University of Oxford, Paediatrics; University of Oxford, Said Buisness School<br>Carter, Alison; University of Oxford, Paediatrics<br>Wells, Glenn; Oxford Academic Health Science Centre<br>Car, Josip; Imperial College London, Primary Care and Social Medicine

**Primary Subject Heading**:	Medical education and training
Secondary Subject Heading:	Qualitative research, Evidence based practice, Research methods, Public health
Keywords:	Massive Open Online Course, Real World Data, Real World Evidence, Continuing education, Data science, Information science

SCHOLARONE™
Manuscripts

Protocol for a Mixed-Methods Evaluation of a Massive Open Online Course on Real World Evidence

Edward Meinert, Abrar Alturkistani, David Brindley, Alison Carter, Glenn Wells, Josip Car

Department of Paediatrics, University of Oxford, OX3 9DU Oxford, United Kingdom Edward Meinert Sir David Cooksey Fellow in Healthcare Translation Global eHealth Unit, Department of Public Health and Primary Care, School of Public Health, Imperial College London, W6 8RP London, United Kingdom Edward Meinert Honorary Research Fellow Department of Infectious Disease Epidemiology, Faculty of Medicine, School of Public Health, Imperial College London, W6 8RP London, United Kingdom Abrar Alturkistani Research Assistant Department of Paediatrics, University of Oxford, OX3 9DU Oxford, United Kingdom David Brindley Senior Research Fellow Department of Paediatrics, University of Oxford, OX3 9DU Oxford, United Kingdom Alison Carter Research Associate Oxford Academic Health Science Centre, OX4 4GA Oxford, United Kingdom Glenn Wells Chief Operating Officer Global eHealth Unit, Department of Public Health and Primary Care, School of Public Health, Imperial College London, W6 8RP London, United Kingdom Josip Car Director of the Global eHealth Unit

Correspondence to: Edward Meinert edward.meinert@paediatrics.ox.ac.uk, e.meinert14@imperial.ac.uk 00447824446808

Word Count:

Keywords: Massive Open Online Course; Real World Evidence; Real World Data; Information Science; Continuing education; Data science

For peer review only

ABSTRACT

Introduction

Increasing number of Massive Open Online Courses (MOOCs) are being used to train learners at scale in various healthcare related skills. However, many challenges in course delivery require further understanding, for example, factors exploring the reasons for high MOOC dropout rates, recorded low social interaction between learners and the lack of understanding of the impact of a course facilitators' presence in course engagement. There is a need to generate further evidence to explore these detriments to MOOC course delivery to enable enhanced course learning design. The proposed mixed-methods evaluation of the MOOC was determined based on the MOOC's aims and objectives and the methodological approaches used to evaluate this type of a course. The MOOC evaluation will help appraise the effectiveness of the MOOC in delivering its intended objectives. This protocol aims to describe the design of a study evaluating learners knowledge, skills and attitudes in a Massive Open Online Course (MOOC) about data science for healthcare.

Methods and Analysis

Study participants will be recruited from learners who have registered for the MOOC. On registration, learners will be given an opportunity to opt into the study and complete informed consent. Following completion of the course, study participants will be contacted to complete semi-structured interviews. Interviews will be transcribed and coded using thematic analysis, with data analysed using two evaluation models: 1) The RE-AIM framework and the 2) Kirkpatrick model drawing data from pre and post-course surveys and post-MOOC semi-structured interviews. The primary goal of the evaluation is to appraise participants' knowledge, skills, and attitude after taking the MOOC.

Ethics and dissemination

Ethics approval for this study was obtained from Imperial College London through the Education Ethics Review Process (EERP) (EERP1617-030). A summary of the research findings will be reported through a peer-reviewed journal and will be presented at an international conference.

For peer review only - http://bmjopen.bmj.com/site/about/guidelines.xhtml

STRENGTHS AND LIMITATIONS OF THIS STUDY

- One strength of the study is the use of qualitative data from semi-structured interviews triangulated via evidence experiences to validate reported activity
- A limitation of the study is the course evaluation may be affected by factors other than the course that may be difficult to identify
- The evaluation of the course is dependent on participant study recruitment via the course

INTRODUCTION

Although research about Massive Open Online Courses (MOOCs) have been increasing with their continued and increased popularity, there remain gaps in understanding on how to achieve similar course impact as compared to face to face instruction.[1] There is a need for evidence on MOOCs to determine how to resolve numerous challenges that impede MOOC uptake and completion.[2] The factors impacting MOOCs delivery can be summarised as 1) low course completion rates (less than 10%) relative to the number of learners who sign up,[3] with more recent evaluation of the literature (2017) revealing that high dropout rates remain a challenge for most MOOCs.[1] 2) lack of social interaction between learners raises concerns about the open and diverse environments that MOOCs generally should offer.[4] 3) Not understanding how the role an educator or facilitator in a MOOC plays in promoting interaction and networking for learners.[5] Further research on how a MOOC can address these issues is needed to facilitate more effective MOOC course design in the future.

Evaluating MOOCs to test their effectiveness is essential for the effective delivery of learning and for appraising its performance. This study will use a mixed-methods combining the RE-AIM framework and the Kirkpatrick evaluation models to evaluate learners' acquisition of learning and skills post-MOOC, and their attitudes towards the course. This evaluation will be of value to all stakeholders involved including the learning institution that developed the course (Imperial College and the University of Oxford), other researchers and learners. The value of the outcomes of the research is understanding how successful the MOOC was to achieving impact to justify further continuing the MOOC and enhancing delivery. Gathering first-hand information on how participants valued the MOOC, their reasons for undertaking the MOOC, and the perceived impact it has had on their studies or working environment or professional practice since this is key in understanding if the MOOC had an impact on participants and is justified for further investment. The value for other researchers will stem from the addition to the literature about MOOCs and MOOC evaluations. The value to students result from the evaluation being used to improve the redesign of the current MOOC and potential improvement of other MOOCs in general based on the insights from this study.

Increased use of data analytics can significantly improve quality and value of health services through increased efficiency and effectiveness.[6] For example, the use of aggregate population level data collection, as implemented in Real World Data (RWD) approaches, could contribute to advancing

For peer review only

capabilities in personalising care, through adjustments in interventions based on real-time analysis of patient responses,[7] and could develop predictive capabilities that can help identify patients at higher risk and contribute to adverse events.[8] For organisations to receive the full benefits of data analytics, there is a growing demand for training staff in data analysis and "to equip managers and employees with relevant professional competencies."[8] MOOCs have been successful in delivering new skills. For example, a MOOC on antimicrobial stewardship in low and middle-income countries reported that 49% of the participants interviewed six months after the MOOC (n=409) have assured that they have implemented the interventions learned from the MOOC in their practice.[9] In addition, a Randomised Controlled Trial found that both MOOCs and a self-paced online educational module were useful in training physiotherapist about spinal cord injuries and increasing their confidence about administering therapy to patients.[10] MOOCs are seen as a suitable method for delivering continuing education in improving their patient care.[11]

This study examines a MOOC centred on Real World Evidence (RWE). The objective of the MOOC is to introduce learners to data analysis methods and techniques of RWE. The MOOC aims to raise awareness of the potential impact RWD data science methods can have on medicine. To evaluate the success of achieving the MOOCs objectives, this study will evaluate the MOOC's ‘reach' of its intended audience and social networks, ‘efficacy' regarding the knowledge/skill gain skill and attrition, and adoption and sustainability of social networks for continual learning in this emerging field; further details of the course instructional design are defined in a separate publication.[12] The evaluation is conducted not only to contribute to the current literature, but also to enable evidence to support future iterations of the course to increase its impact.

Research Question

The primary research question of the evaluation is: How has the course impacted learners' knowledge, skills and attitudes on the use of data science in healthcare?

The secondary questions of the evaluation include the following:

- What evidence is there that the intended target audience was reached?
- What evidence is there that the MOOC has made a difference to participants in their work or studies
- What evidence is there of participant networks for data science in healthcare being adopted during the MOOC?
- What evidence is there that the MOOC format and materials engaged participants?
- What evidence is there of participant networks for data science in healthcare being sustained post--MOOC?

METHODS AND ANALYSIS

Study participants will be recruited from learners who have registered for the MOOC. On registration to the course, learners will be given an opportunity to opt into the study, receiving a participant information sheet (Appendix 1). Study participants will also be recruited via email (Appendix 2). Should learners wish to participate, they will sign an informed consent form (Appendix 3). Following completion of the course, study participants will be contacted to schedule interviews. A researcher

For peer review only - http://bmjopen.bmj.com/site/about/guidelines.xhtml

holding post-graduate level training in qualitative research methods (via Imperial College's Master of Public Health and/or Doctorate training programme in Clinical Medicine Research) will hold semi-structured interviews with the study participant (Appendix 4).

Study Design

This study will apply two evaluation methods to investigate the impact of the MOOC. The RE--AIM framework will be used to evaluate the reach, delivery (implementation) and sustainability (maintenance) of the MOOC with efficacy and adoption examined by the Kirkpatrick Model. The Kirkpatrick evaluation will follow the four levels of assessment: reaction, learning, behaviour and results, [13] where a particular focus will be given to determine if participants were able to increase their learning through the course, if they were able to apply the skills learned in the course in their study/workplace (adoption) and if through attending the MOOC they were able to influence their broader community (efficacy). The RE--AIM framework will be used for evaluation of reach at the participant level. We will examine total recruitment on the course and compare their characteristics to eligibility criteria, demographic information and other measures. Facilitators and barriers to individual patient recruitment and suggestions for improvement will be identified through interviews with the research team. Evaluation of the implementation will be done at participant level via a post-course survey (for graded feedback on course delivery to include materials, content, layout and format, etc.) and post-course interviews to discuss perceptions of participants in greater depth. Maintenance will be evaluated at participant level to measure the continuation of MOOC effects over time. This will be conducted via a post-course interview, held three months post-course. For specific examples and evidence to substantiate participants views/claims To evaluate impact further, the Kirkpatrick evaluation model shall be used. For Level 1 (Reaction), the survey material completed pre and post-course will be captured. For learners who did not complete immediate post--course surveys, this will be noted, but reflections of the course will be captured in the interview (for recording purposes). For Level 2 (Learning), the learning record from the MOOC shall be used. For Level 3 (Behaviour), the semi- structured interviews will investigate the impact of the MOOC on multiple factors of professional behaviour. For Level 4 (Results), through aggregation and coding of the interview results, the research team will analyse the overall impact of the results on training. [13] The Kirkpatrick model was selected for evaluating the MOOC due to it being directed towards evaluating training programmes designed for professional development training,[13] and since this MOOC was designed to influence learners' skills and behaviour, this was seen as a suitable model. Also, the model is commonly used for MOOC evaluations as other studies have reported using the Kirkpatrick model in their evaluation methodologies. [14-16]

Participants

All learners who participated in the MOOC for any length of time will be recruited for the study. Participants recruited will differ due to the diversity of participants joining a MOOC in general. Possible differences will be in the level of data science knowledge and MOOC completion. We will try to reflect this diversity in the participants recruited for the study, by categorising participants and aggregating their results in their response group classification (e.g. undergraduates in data science who completed the post-course survey). An additional high-level classification is all learners who (1) completed the pre-course survey, (2) completed the post- course survey, (3) completed the

For peer review only - http://bmjopen.bmj.com/site/about/guidelines.xhtml

certificate track; (further categories shall be analysed depending on respondents). The exclusion criteria shall exclude learners who are employed by Imperial College London or are known by the researchers, therefore addressing possible power issues.

Recruitment

The participants in this MOOC self--select by registering for the course and participants in this study will be drawn from this pool. All those who have participated in the course will be approached, to prevent participation bias. The learners will be contacted by the research team to participate in the interviews. To avoid conflicts, the exclusion criteria have been designed to avoid any power of coercion from the participants. All learners will be contacted via email twice for participation over a two--week period. This method is consistent with previous contact methods during the course and was selected to be non-intrusive to learners.

Sample Size

We are aiming to recruit 16 learners to the study. We predict that interviews from 16 learners will generate enough data for answering the research questions and fall within the scope of effort allocated for this investigation, additionally recent literature suggests that this amount of thematic data will be sufficient for qualitative analysis. [17] However, if there is a need for further investigation of further phenomena or themes, we will endeavour to get more participants into being interviewed for the study. Participants will be interviewed until saturation is reached for all the key themes about course effectiveness.

Data Collection

Pre and post-course surveys

Pre and post-course surveys are surveys that are administered online and that are accessible to all learners who have participated in the MOOC. The surveys capture the learners' general reaction to the course. Pre-course surveys include questions about reasons for taking the course, preferred learning methods, and current knowledge of the topic being taught. The post-course survey gives the learners a chance to provide graded feedback on course delivery to including feedback on the materials provided, the content of the course, and the design of the MOOC (layout and format). The delivery of the post-course survey will be segmented cross-sectionally by learner groups identified from course trends (e.g. those who completed the course, those who only participated in part of the course, those who registered but did not complete a significant portion of the course). Survey results will be structured on a Likert scale, using a Kruskal-Wallis test to identify comparison of data between groups. Logistic regression analysis will determine statistically significant results among groups. Reporting structure for the surveys are reported in a strengthening the reporting of observational studies (STROBE) statement [18], which is detailed in Appendix 5.

Semi-structured interviews

Interviews are scheduled to take place between 30 to 60 minutes (maximum). The interviews will be executed at greater than three months than the course execution to allow for analysis of the impact

For peer review only - http://bmjopen.bmj.com/site/about/guidelines.xhtml

of the course has had on behaviour. Interviews will be conducted through Skype and telephone conference calls because course participants are distributed globally, and this is the most accessible means of interviewing participants. [19] All interviews will be recorded. The reason semi-structured interviewing is being used is to investigate further what factors could impact behaviour that is not fully understood at this stage in the study. This will also allow for aggregation of responses and the ability to allow further examination of learners' perspectives on the research question categories. 16 learners will take approx. 160 hours of transcription/analysis to complete with a planned effort of 5 weeks. Interview recordings will be transcribed by an internal third party (an Imperial College staff member trained in transcription) and given to study participants for review for accuracy. Reporting structure for the interviews are reported in the consolidated criterion for reporting qualitative research (COREQ) Statement [20], which is detailed in Appendix 6.

Study Ethics

Anonymisation via a unique ID will be created to protect confidentiality. The primary key between unique ID and participant shall be securely held on a secured drive at Imperial College. Only the research administrators will have access to correlate information to respondents (via a primary key) which will be stored on a secured drive at Imperial College. The reason the primary key is being maintained is in the event of participant wishes to withdraw their data from the study; should a request be received all of their corresponding data and files shall be destroyed. Only the research administrators shall have access to this file. The British Educational Research Association (BERA) guidelines, [21] have been followed for voluntary informed consent use of methods and voluntary informed consent, use of methods and departmental policies in the event there are issues in delivery. All participants will receive an information sheet with adequate reading time, and all participants will be asked to sign a written informed consent explaining that all participants have the right to withdraw and remove their data should they decide to even after the interview has been completed. To reimburse participants for their time in participating in the interviews, three response participants will be randomly selected to receive a 40-pound voucher from amazon.co.uk. This study will not include children, vulnerable young people or vulnerable adults. If there are problems raised during the study, this will be escalated to the Head of the Department who will act following discussion with the PI.

Data Analysis

Data analysis will be performed using thematic analysis methods and then evaluate the responses based on the RE-AIM framework and Kirkpatrick evaluation models. The RE-AIM framework has been utilised because of its recognition to identify adoption trends [22], while the Kirkpatrick method will form a data set for triangulation. Coding of responses will be completed by an independent review of transcripts (by two members of staff) to ensure consistency in analysis.

Patient and Public Involvement

Members of the public informed the development of research questions and study objectives via a workshop held at the European Scientific Institute in July 2017. Learners participating in this study completed informed consent (Appendix 3) and shall receive a copy of results and publications from this work (Appendix 1).

For peer review only - http://bmjopen.bmj.com/site/about/guidelines.xhtml

ETHICS AND DISSEMINATION

Ethics approval for this study was obtained from Imperial College London through the Education Ethics Review Process (EERP) (EERP1617-030). A report summarising the research findings will be published in a peer-reviewed journal. A presentation will be given to a selected audience of health professionals and academics, to include individuals from Imperial College. Findings will also be presented at an international conference.

Acknowledgements

This work was funded by EIT Health (Grant 18654). This work was also supported by the Sir David Cooksey Fellowship in Healthcare Translation, the Oxford Academic Health Sciences Centre and the SENS Research Foundation.

Contributorship Statement

EM conceived the evaluation objectives and methods. AA and EM drafted the design and development of the protocol. AA and EM then completed the first draft of the manuscript. DB, AC, GW and, JC reviewed the second draft. AA and EM incorporated and addressed the feedback from the authors. EM responded to all external peer review comments. All authors read and approved the final manuscript. All authors completed the ICMJE uniform disclosure form at www.icmje.org/coi_disclosure.pdf. There are no relevant conflicts of interest, financial or other types of relationships that may influence the manuscript declared by authors. Authors do not have any patents and are not associated to any conditions or circumstances that may lead to conflicts of interest.

Conflicts of Interest

The ICMJE uniform disclosure form at http://www.icmje.org/conflicts-of-interest/ was completed by all authors. All authors declare that they have no relevant conflicts of interest, no financial relationships that could potentially influence the submitted manuscript. All authors do not have any patents that may be relevant to the manuscript or any relationship, circumstances or conditions that may lead to conflicts of interest. This article represents the authors' individual opinions and may not necessarily represent the viewpoints of their employers. D.B. is a stockholder in Translation Ventures. Ltd. (Charlbury, Oxfordshire, UK) and IP Asset Ventures Ltd. (Oxford, Oxfordshire, UK), companies that, among other services, provide cell therapy biomanufacturing, regulatory, and financial advice to pharmaceutical clients. D.B. is also subject to the CFS institute's codes, standards, and guidelines, so he must stress that this piece is provided for academic interest only and must not be seen in any way as an investment recommendation.

Funding statement

This work was funded by EIT Health (Grant 18654).

For peer review only - http://bmjopen.bmj.com/site/about/guidelines.xhtml

For peer review only

For peer review only - http://bmjopen.bmj.com/site/about/guidelines.xhtml

References

1. Bozkurt A, Akgün-Özbek E, Zawacki-Richter O. Trends and Patterns in Massive Open Online Courses: Review and Content Analysis of Research on MOOCs (2008-2015). *The International Review of Research in Open and Distributed Learning* 2017;**18**. doi:10.19173/irrodl.v18i5.3080
2. Sinclair J, Boyatt R, Rocks C, *et al.* Massive open online courses : a review of usage and evaluation. *International journal of learning technology* 2015;**10**:1–23.
3. Khalil H, Ebner M. MOOCs Completion Rates and Possible Methods to Improve Retention - A Literature Review. Association for the Advancement of Computing in Education (AACE) 2014. 1305–13.https://www.learntechlib.org/primary/p/147656/ (accessed 2 Jul 2018).
4. Tawfik AA, Reeves TD, Stich AE, *et al.* The nature and level of learner–learner interaction in a chemistry massive open online course (MOOC). *J Comput High Educ* 2017;**29**:411–31. doi:10.1007/s12528-017-9135-3
5. Goldie JGS. Connectivism: A knowledge learning theory for the digital age? *Med Teach* 2016;**38**:1064–9. doi:10.3109/0142159X.2016.1173661
6. Bates DW, Heitmueller A, Kakad M, *et al.* Why policymakers should care about “big data” in healthcare. *Health Policy and Technology* 2018;**7**:211–6. doi:10.1016/j.hlpt.2018.04.006
7. Firouzi F, Rahmani AM, Mankodiya K, *et al.* Internet-of-Things and big data for smarter healthcare: From device to architecture, applications and analytics. *Future Generation Computer Systems* 2018;**78**:583–6. doi:10.1016/j.future.2017.09.016
8. Wang Y, Kung L, Byrd TA. Big data analytics: Understanding its capabilities and potential benefits for healthcare organizations. *Technological Forecasting and Social Change* 2018;**126**:3–13. doi:10.1016/j.techfore.2015.12.019
9. Sneddon J, Barlow G, Bradley S, *et al.* Development and impact of a massive open online course (MOOC) for antimicrobial stewardship. *J Antimicrob Chemother* 2018;**73**:1091–7. doi:10.1093/jac/dkx493
10. Hossain MS, Shofiqul Islam M, Glinsky JV, *et al.* A massive open online course (MOOC) can be used to teach physiotherapy students about spinal cord injuries: a randomised trial. *Journal of Physiotherapy* 2015;**61**:21–7. doi:10.1016/j.jphys.2014.09.008
11. Pickering JD, Swinnerton BJ. An Anatomy Massive Open Online Course as a Continuing Professional Development Tool for Healthcare Professionals. *MedSciEduc* 2017;**27**:243–52. doi:10.1007/s40670-017-0383-7
12. Meinert E, Alturkistani A, Car J, *et al.* Real World Evidence for postgraduate students and professionals in healthcare: protocol for the design of a blended Massive Open Online Course. *BMJ Open.*
13. Kirkpatrick DL. *Evaluating training programs: the four levels*. 3rd ed. San Francisco, CA: Berrett-Koehler 2005.

For peer review only

14. Lin J, Cantoni L. Assessing the Performance of a Tourism MOOC Using the Kirkpatrick Model: A Supplier's Point of View. *Information and Communication Technologies in Tourism 2017* 2017;:129–42. doi:10.1007/978-3-319-51168-9_10
15. Ayub E, Wei GW, Yue WS. Exploring Factors Affecting Learners' Acceptance of MOOCs Based on Kirkpatrick's Model. In: *Proceedings of the 8th International Conference on E-Education, E-Business, E-Management and E-Learning*. New York, NY, USA: : ACM 2017. 34–39. doi:10.1145/3026480.3026490
16. Goh WW, Wong SY, Ayub E. The Effectiveness of MOOC Among Learners Based on Kirkpatrick's Model. *Redesigning Learning for Greater Social Impact* 2018;:313–23. doi:10.1007/978-981-10-4223-2_29
17. Saunders MNK, Townsend K. Reporting and Justifying the Number of Interview Participants in Organization and Workplace Research. *British Journal of Management*;**27**:836–52. doi:10.1111/1467-8551.12182
18. von Elm E, Altman DG, Egger M, Pocock SJ, Gøtzsche PC, Vandenbroucke JP; STROBE Initiative. The Strengthening the Reporting of Observational Studies in Epidemiology (STROBE)statement: guidelines for reporting observational studies. *Lancet* 2007 Oct 20;370(9596):1453-7. doi: https://doi.org/10.1016/S0140-6736(07)61602-X
19. Janghorban R, Roudsari RL, Taghipour A. Skype interviewing: The new generation of online synchronous interview in qualitative research. *Int J Qual Stud Health Well-being* 2014;**9**. doi:10.3402/qhw.v9.24152
20. Allison Tong, Peter Sainsbury, Jonathan Craig; Consolidated criteria for reporting qualitative research (COREQ): a 32-item checklist for interviews and focus groups, *International Journal for Quality in Health Care*, Volume 19, Issue 6, 1 December 2007, Pages 349–357, https://doi.org/10.1093/intqhc/mzm042
21. British Education Research Association. BERA Ethical Guidelines for Educational Research 2011. Researchers' Resources. 2013.https://www.bera.ac.uk/researchers-resources/publications/bera-ethical-guidelines-for-educational-research-2011 (accessed 2 Jul 2018).
22. Patton MQ. Enhancing the quality and credibility of qualitative analysis. *Health Serv Res* 1999;**34**:1189–208.

For peer review only - http://bmjopen.bmj.com/site/about/guidelines.xhtml

APPENDICES

Appendix 1: Information sheet for participants

INFORMATION SHEET FOR PARTICIPANTS

YOU WILL BE GIVEN A COPY OF THIS INFORMATION SHEET.

Data Science Essentials MOOC Evaluation (in Real World Evidence)

Name of researcher: Josip Car MD PhD

We would like to invite you to participate in this research project. You should only participate if you want to; choosing not to take part will not disadvantage you in any way.

Before you decide whether you want to take part, it is important for you to understand why the research is being done and what your participation will involve. Please take time to read the following information carefully and discuss it with others if you wish. Ask us if there is anything that is not clear or if you would like more information.

1. Project aims and rationale

The aim of this study is to establish the perceived impact this form of course delivery had on developing skills in improving healthcare quality and establishing a community of individuals with similar subject-matter interests. The study is to evaluate and explore the factors that may have influenced change in behaviour in professional skills following completion of the course.

By taking part in the study, you will allow the research team to better understand the effectiveness of the course and make recommendations on how to improve the course in subsequent iterations.

2. Participant inclusion and exclusion criteria

The study aims involve 16 participants who have participated in the Data Science Essentials course in Real World Evidence, but this excludes any individual who is employed by Imperial College London or who has professional/personal relationship with Dr Josip Car, Edward Meinert, Mel Toumazos or Tasnime Osama. Should more than 16 indicate interest in partaking, participants will be selected in order to represent as many different working backgrounds and different levels of course completion as possible.

3. Process of participation

It is up to you to decide whether you want to take part. If you choose to participate, you will be given this information sheet to keep and you will be asked to sign a consent form. If you choose to take part in the study, you are still free to withdraw at any time and without giving a reason, and you will be given the option to have your data destroyed. A decision to withdraw at any time, or a decision not to take part, will not impact you in any way.

You will participate in Skype video conference or telephone conference for 30 to 60 minutes where you will be asked interview questions relating to the course and how it may have impacted your

For peer review only - http://bmjopen.bmj.com/site/about/guidelines.xhtml

professional practice since taking the course. The sessions shall be recorded by the researchers investigating the impact of the course, subject to your permission, and transcribed by a 3rd party at Imperial College. If you are a member of NHS staff you will require line management approval to participate in this study.

4. Risks to participants

There is a risk that participants may be impacted negatively in terms of the cost to their time that participation would require, therefore the interviews will be completed in less than 60 minutes. The interviews will be transcribed by an internal third party in Imperial and information will not be disclosed externally.

5. Process for ensuring anonymity and confidentiality

If you consent to take part in the research, any information you provide may be inspected and used by administrators of the study.

Each participant will be anonymised using a unique identifier to maintain confidentiality and all data will be securely stored and managed according to Imperial College rules and expected practices.

Raw, un-anonymised audio data will be securely stored separately from the anonymisation key and deleted when it is no longer needed. The anonymised transcripts will be securely stored according to Imperial College protocols and regulations

6. Access to information

All information which is collected about you during the study will be strictly confidential and accessible only by Imperial College study administrators. Any information which leaves the research administration will have your personal information removed so that you cannot be recognised from it.

7. Possible benefits to participation

By taking part in the study, you will allow the research team to better understand the effectiveness of the course and make recommendations on how to improve the course in subsequent iterations. Additionally, all of those who participate in the interviews will be entered into a prize draw, and three will be randomly selected to receive a £40 (or currency equivalent) Amazon voucher.

8. Plans for dissemination

A report summarising the findings will be published in a peer reviewed journal.

A copy of the study information sheet, along with a copy of the consent form will be provided to you, should you choose to take part in the study.

If you have any questions or require more information about this study, please contact the co-researcher using the following contact details:

Edward Meinert

Global eHealth Unit

Department of Primary Care and Public Health, School of Public Health, Imperial College London

For peer review only - http://bmjopen.bmj.com/site/about/guidelines.xhtml

The Reynolds Building | St Dunstan's Road| London W6 8RP | UK

Email: e.meinert14@imperial.ac.uk | Telephone: +44 (0)782 444 6808.

Thank you for taking the time to read and consider this information.

For peer review only

For peer review only - http://bmjopen.bmj.com/site/about/guidelines.xhtml

Appendix 2: Sample Recruitment Email

Dear Participant,

Thank you for your interest in the Data Science Essentials course in Real World Evidence.

To evaluate the course, we are looking for participants who are interested in having a phone interview or skype discussion about their experience of the MOOC and the effect is has had on their working environment or academic advancement. The interview will take from 30 to 60 minutes. To compensate you for your time, three participants will be randomly entered into a draw to receive a £40 voucher for amazon.co.uk (around a 1 in 5 chance).

If you have any questions, please send them to gehu-training@imperial.ac.uk

If you would like to register your interest, please click the link below:

(link to survey monkey)

For peer review only

01/01/2018
Version 3.0

Appendix 3: Informed Consent Form

INFORMED CONSENT FORM

Title of Project: Data Science Essentials (in Real World Evidence) MOOC Evaluation

Name of researcher: Josip Car MD PhD

Please initial box.

1. I confirm that I have read and understand the subject information sheet dated 31/06/2017 version 1.0 for the above study. I may keep this information sheet for my records and I have had the opportunity to ask questions which have been answered fully.

2. I understand that my participation is voluntary and I am free to withdraw, without giving any reason and without being penalised or disadvantaged in any way.

3. I understand that sections of my recorded comments and transcript text may be looked at by responsible individuals from Imperial College London. I give permission for these individuals to access this data as relevant to this and future research.

4. I am willing to have this interview recorded.

5. I understand that this consent form will be kept separate from the data and that the researchers will maintain my anonymity throughout the project, including in publication.

6. I confirm that I am not employed by Imperial College London or have a personal/professional relationship with Dr Josip Car, Edward Meinert, Mel Toumazos or Tasnime Osama

7. I agree to take part in the above study.

* *Delete as appropriate*

Name of Participant (Printed)	Date	Signature

Name of Researcher	Date	Signature

Version 3.0
Date: 01/01/2018

For peer review only - http://bmjopen.bmj.com/site/about/guidelines.xhtml

For peer review only

(Printed)

1 copy for subject; 1 copy for researcher.

For peer review only

For peer review only - http://bmjopen.bmj.com/site/about/guidelines.xhtml

Appendix 4: Sample interview questions

- Participant background
  - Current degree course/ working role
  - Education
  - Experience of healthcare
  - Experience of data science methodologies and tools
  - Experience in taking part in MOOCs
  - Experience of using social media.

- Participant's need for a MOOC in Data Science focusing on healthcare data.

  This section will have a series of follow up questions with opportunity for in depth discussion

  - Does this meet a current a need?
  - Have they encountered any barriers to learning in this field?
  - How did this method of delivery (the MOOC) meet their needs?
  - What have they come across which already exists to meet this need?
- Why participant signed-up to the MOOC.

  - The expectations and aims of participants

  This section will cover the expectations of participants when joining the course, and what they aimed to take from it

- How much of MOOC had individual completed?

  Follow up questions:
  - More or less than hoped?
  - What factors helped and hindered this?
  - What modules did they complete?
  - If not finished, did they intend to?

01/01/2018
Version 3.0

For peer review only

- Social media and creating online learning communities
  Exploring whether the interviewee collaborates and learns via MOOC networks
  - Did they join in during the course? What were their thoughts?
  - Are they still connected with other MOOC learners via networks?
- What did individuals enjoy about the MOOC and why?
  - Views on content, materials and format.

- What could be improved or done differently?
  Follow up question:
  - Any technical issues?

- Value for participant
  - What value did you take from having done the MOOC?
  - How valuable was the networking aspect of the MOOC?
  - Do you have specific examples of data science projects/work that you have implemented or been involved in since undertaking the MOOC?
  - How likely are these projects to have happened if you had not participated in the MOOC?

  Potential follow up:
  - Enquiring as to whether this was in line with their original expectation, and why it may have differed

- Application of learning
  - Have participants been able to apply learning from the MOOC?
  - Do they have plans to apply in the future?
  - How has it affected them in a day-to-day way?

- Are they planning of applying for certification?
  Follow up:
  Why or why not?

For peer review only - http://bmjopen.bmj.com/site/about/guidelines.xhtml

Appendix 5: STROBE Statement

STROBE Statement—Checklist of items that should be included in reports of ***cross-sectional studies***

	Item No	Recommendation
**Title and abstract**	1	(*a*) Indicate the study's design with a commonly used term in the title or the abstract<br>**Page 1 – Title**
		(*b*) Provide in the abstract an informative and balanced summary of what was done and what was found<br>**Page 2 – Abstract**
**Introduction**		
Background/rationale	2	Explain the scientific background and rationale for the investigation being reported<br>**Page 3 – Introduction, Page 4 – Research Question**
Objectives	3	State specific objectives, including any prespecified hypotheses<br>**Page 3 – Introduction, Page 4 – Research Question; No prespecified hypotheses as not experimental study; evaluation study on learning impact**
**Methods**		
Study design	4	Present key elements of study design early in the paper<br>**Page 4 – Methods, Page 5 – Study design**
Setting	5	Describe the setting, locations, and relevant dates, including periods of recruitment, exposure, follow-up, and data collection<br>**Page 5 – Participants, Page 6 – Recruitment, Page 6,7 – Data collection**
Participants	6	(*a*) Give the eligibility criteria, and the sources and methods of selection of participants<br>**Page 5 – Participants**
Variables	7	Clearly define all outcomes, exposures, predictors, potential confounders, and effect modifiers. Give diagnostic criteria, if applicable<br>**Page 5 – Participants**
Data sources/ measurement	8*	For each variable of interest, give sources of data and details of methods of assessment (measurement). Describe comparability of assessment methods if there is more than one group<br>**Page 6,7 – Data collection**
Bias	9	Describe any efforts to address potential sources of bias<br>**Page 6 – Recruitment**
Study size	10	Explain how the study size was arrived at<br>**Page 6 – Sample size**
Quantitative variables	11	Explain how quantitative variables were handled in the analyses. If applicable, describe which groupings were chosen and why<br>**Page 6 – Pre and post-course surveys**
Statistical methods	12	(*a*) Describe all statistical methods, including those used to control for confounding
		(*b*) Describe any methods used to examine subgroups and interactions
		(*c*) Explain how missing data were addressed
		(*d*) If applicable, describe analytical methods taking account of sampling strategy
		(*e*) Describe any sensitivity analyses<br>**Page 6 – Pre and post course-surveys**

For peer review only

**Results**		
Participants	13*	(a) Report numbers of individuals at each stage of study—eg numbers potentially eligible, examined for eligibility, confirmed eligible, included in the study, completing follow-up, and analysed
		(b) Give reasons for non-participation at each stage
		(c) Consider use of a flow diagram<br>**Page 5 – Participants**
Descriptive data	14*	(a) Give characteristics of study participants (eg demographic, clinical, social) and information on exposures and potential confounders
		(b) Indicate number of participants with missing data for each variable of interest<br>**Page 5 – Participants**
Outcome data	15*	Report numbers of outcome events or summary measures<br>**N/A – Study to be executed**
Main results	16	(*a*) Give unadjusted estimates and, if applicable, confounder-adjusted estimates and their precision (eg, 95% confidence interval). Make clear which confounders were adjusted for and why they were included
		(*b*) Report category boundaries when continuous variables were categorized
		(*c*) If relevant, consider translating estimates of relative risk into absolute risk for a meaningful time period<br>**N/A – Study to be executed**
Other analyses	17	Report other analyses done—eg analyses of subgroups and interactions, and sensitivity analyses<br>**N/A – Study to be executed**
**Discussion**		
Key results	18	Summarise key results with reference to study objectives<br>**N/A – Study to be executed**
Limitations	19	Discuss limitations of the study, taking into account sources of potential bias or imprecision. Discuss both direction and magnitude of any potential bias<br>**N/A – Study to be executed**
Interpretation	20	Give a cautious overall interpretation of results considering objectives, limitations, multiplicity of analyses, results from similar studies, and other relevant evidence<br>**N/A – Study to be executed**
Generalisability	21	Discuss the generalisability (external validity) of the study results<br>**N/A – Study to be executed**
**Other information**		
Funding	22	Give the source of funding and the role of the funders for the present study and, if applicable, for the original study on which the present article is based<br>**Page 8 – Funding statement**

*Give information separately for exposed and unexposed groups.

Note: An Explanation and Elaboration article discusses each checklist item and gives methodological background and published examples of transparent reporting. The STROBE checklist is best used in conjunction with this article (freely available on the Web sites of PLoS Medicine at http://www.plosmedicine.org/, Annals of Internal Medicine at http://www.annals.org/, and Epidemiology at http://www.epidem.com/). Information on the STROBE Initiative is available at www.strobe-statement.org.

For peer review only - http://bmjopen.bmj.com/site/about/guidelines.xhtml

Appendix 6: COREQ Checklist

Manuscript: Protocol for a Mixed-Methods Evaluation of a Massive Open Online Course on Real World Evidence

Edward Meinert, Abrar Alturkistani, David Brindley, Alison Carter, Glenn Wells, Josip Car

Consolidated criteria for reporting qualitative studies (COREQ): 32-item checklist

Developed from:
Tong A, Sainsbury P, Craig J. Consolidated criteria for reporting qualitative research (COREQ): a 32-item checklist for interviews and focus groups. *International Journal for Quality in Health Care*. 2007. Volume 19, Number 6: pp. 349 – 357

No. Item	Guide questions/description	Reported on Page #
**Domain 1: Research team and reflexivity**		Page 1
Personal Characteristics		
1. Inter viewer/facilitator	Which author/s conducted the inter view or focus group?	Appendix 1
2. Credentials	What were the researcher's credentials? E.g. PhD, MD	Pages 4-5, Appendix 1
3. Occupation	What was their occupation at the time of the study?	Pages 4-5
4. Gender	Was the researcher male or female?	Appendix 1
5. Experience and training	What experience or training did the researcher have?	Pages 4-5
Relationship with participants		
6. Relationship established	Was a relationship established prior to study commencement?	Page 4 .
7. Participant knowledge of the interviewer	What did the participants know about the researcher? e.g. personal goals, reasons for doing the research	Appendix 1
8. Interviewer characteristics	What characteristics were reported about the inter viewer/facilitator? e.g. Bias, assumptions, reasons and interests in the	Page 6

For peer review only

	research topic	

**Domain 2: study design**		
Theoretical framework		
9. Methodological orientation and Theory	What methodological orientation was stated to underpin the study? e.g. grounded theory, discourse analysis, ethnography, phenomenology, content analysis	Page 5
Participant selection		
10. Sampling	How were participants selected? e.g. purposive, convenience, consecutive, snowball	Page 6
11. Method of approach	How were participants approached? e.g. face-to-face, telephone, mail, email	Page 6
12. Sample size	How many participants were in the study?	Page 6
13. Non-participation	How many people refused to participate or dropped out? Reasons?	N/A – study is to occur
Setting		
14. Setting of data collection	Where was the data collected? e.g. home, clinic, workplace	Page 6<br>.
15. Presence of non-participants	Was anyone else present besides the participants and researchers?	Page 6
16. Description of sample	What are the important characteristics of the sample? e.g. demographic data, date	Page 6
Data collection		
17. Interview guide	Were questions, prompts, guides provided by the authors? Was it pilot tested?	Appendix 4
18. Repeat interviews	Were repeat inter views carried out? If yes, how many?	N/A – study is to occur
19. Audio/visual recording	Did the research use audio or visual recording to collect the data?	Page 6

For peer review only

20. Field notes	Were field notes made during and/or after the inter view or focus group?	Page 6
21. Duration	What was the duration of the inter views or focus group?	Page 6
22. Data saturation	Was data saturation discussed?	Page 6
23. Transcripts returned	Were transcripts returned to participants for comment and/or correction?	Page 7
**Domain 3: analysis and findings**		
Data analysis		
24. Number of data coders	How many data coders coded the data?	Page 7
25. Description of the coding tree	Did authors provide a description of the coding tree?	Page 7
26. Derivation of themes	Were themes identified in advance or derived from the data?	Page 7
27. Software	What software, if applicable, was used to manage the data?	N/A – study is to occur
28. Participant checking	Did participants provide feedback on the findings?	N/A – study is to occur
Reporting		
29. Quotations presented	Were participant quotations presented to illustrate the themes/findings? Was each quotation identified? e.g. participant number	N/A – study is to occur
30. Data and findings consistent	Was there consistency between the data presented and the findings?	N/A – study is to occur
31. Clarity of major themes	Were major themes clearly presented in the findings?	Page 7
32. Clarity of minor themes	Is there a description of diverse cases or discussion of minor themes?	Page 7

For peer review only
