# Supplementary material for: Protocol for a mixed-methods evaluation of a massive open online course on real world evidence
Source: BMJ Open. 2018 Aug 13;8(8):e025188. doi: 10.1136/bmjopen-2018-025188 (PMC6091905; doi:10.1136/bmjopen-2018-025188)
Supplement: Reviewer comments [file bmjopen-2018-025188.reviewer_comments.pdf]

PEER REVIEW HISTORY

BMJ Open publishes all reviews undertaken for accepted manuscripts. Reviewers are asked to complete a checklist review form (**http://bmjopen.bmj.com/site/about/resources/checklist.pdf**) and are provided with free text boxes to elaborate on their assessment. These free text comments are reproduced below.

ARTICLE DETAILS

**TITLE (PROVISIONAL)**	Protocol for a Mixed-Methods Evaluation of a Massive Open Online Course on Real World Evidence
**AUTHORS**	Meinert, Edward; Alturkistani, Abrar; Brindley, David; Carter, Alison; Wells, Glenn; Car, Josip

This article was not externally reviewed at BMJ Open. Protocols that have been independently assessed prior to submission to BMJ Open are usually fast-tracked to publication on the grounds that further substantial changes will not be possible. This independent assessment will usually be external, independent review for both a substantial grant award from a non-commercial or government funder and ethics approval.
